# Diastematomyelia Presenting With no Pain in a 53-Year-Old Man: A Case Report

**DOI:** 10.5812/ircmj.4195

**Published:** 2013-06-05

**Authors:** Parisa Azimi, Hassan Reza Mohammadi

**Affiliations:** 1 Department of Neurosurgery, Imam Hossain Medical Center, University of Shahid Beheshti Medical Sciences, Tehran, IR Iran

**Keywords:** Diastematomyelia, Pain, Spinal Cord

## Abstract

Initial presentation of diastematomyelia is rarely seen in adults. The purpose of this case report is to present a case of clinically silent diastematomyelia unrecognized into adulthood and review of the literature. A 53-year-old Persian man was admitted to our hospital with gait disturbance, weakness of the right lower extremity, sensory loss of the left and right lower extremity of two weeks’ duration, with no pain or sphincter dysfunction. The patient underwent radiological examinations, and diastematomyelia was diagnosed. The deteriorating condition of our patient led to the decision to perform a surgery. A laminectomy was performed from L-3 to L-5 with resection of the soft-tissue mass and excision of the bony spur, and the patient was followed for 6 months. Postoperatively, the patient did not show new neurologic deficit and he returned to work 4 months after surgery. Our case was unique because of the absence of any pain, neurologic signs, and precipitating acute event leading to diagnosis, until 53 years of age. Surgical decompression of bony spur provided relative improvement of his symptoms.

## 1. Introduction

Diastematomyelia was first described in 1837 by Ollivier (1). It is a rare form of spinal dysraphism characterized by a sagittal cleft of varying extent that splits the spinal cord, conus medullaris, or filum terminale with splaying of the posterior vertebral elements. This condition can be caused by an osseous, cartilaginous, or fibrous septum, producing a complete or incomplete sagittal division of the spinal cord into two hemicords (2). Most patients with diastematomyelia present in childhood and this condition rarely presents in adults ( 3). We are reporting a case of diastematomyelia in adults. The clinical summary, imaging findings, and surgical procedures will be discussed.

## 2. Case presentation

**Figure 1.**
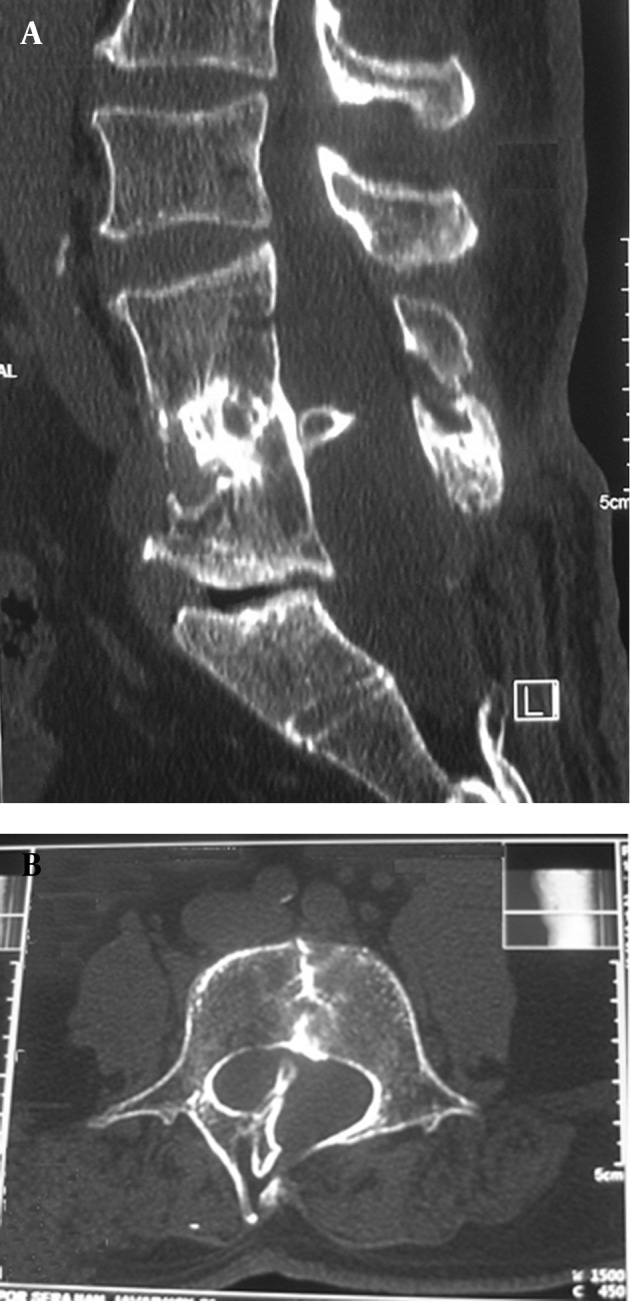
Mid-sagittal (A), and Axial (B) CT Images Showing Bony Spur That Extends to the Posterior Lamina ofL4, Spina Bifida at S1-S2, and Block Vertebra From L3 to L5

**Figure 2.**
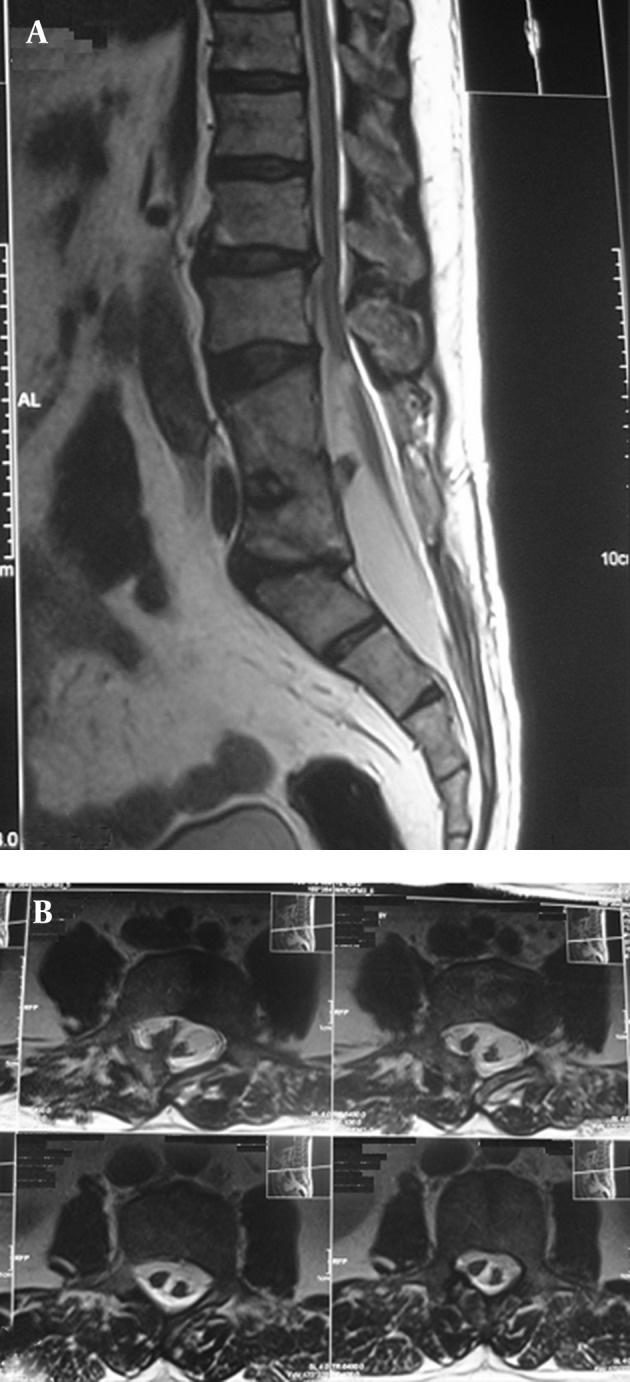
Mid-sagittal (A), and Axial (B) T2-weighted Images Showing Split Spinal Cord. The Spinal Cord is Split From Approximately L3 to L4 With Tethering of the tip of the Conus Medullaris at L5

A 53-year-old Persian man reported suffering from gait disturbance, weakness of the right lower extremity and sensory loss in both lower extremities of two weeks duration, without pain or sphincter dysfunction. The symptoms began after a 150 km driving experience. Motor examination revealed (Medical Research Council (MRC) grading) 4/5 and 2/5 power in the proximal and distal muscle groups of the right leg , respectively. The left lower extremity exhibited normal power. Follow-up computed tomography (CT) and magnetic resonance imaging (MRI) were diagnostic for diastematomyelia. A bony spur was extended to the posterior lamina of L4 and a spina bifida was present at the sacral level (S1-S2) , and block vertebra from L3 to L5 (Figure 1). The spinal cord was split from approximately L-3 to L-4 with tethering of the tip of the conus medullaris at L-5 (Figure 2).

**Figure 3.**
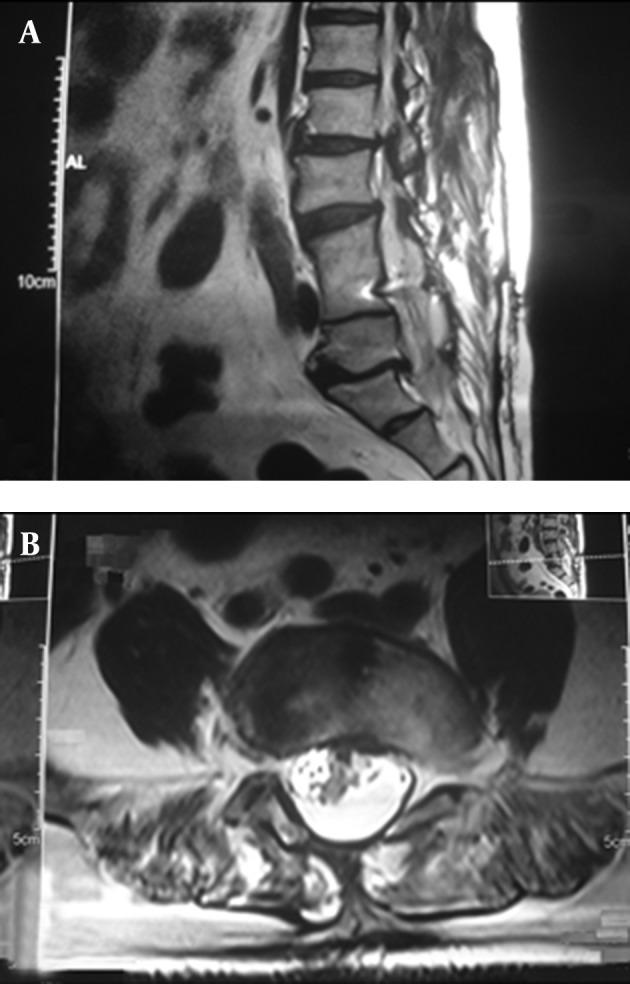
Mid-sagittal (A) and Axial (B) T2-weighted MRI Images Showing Removal of the Bony Spur and Release of the Spinal Cord

The deteriorating condition of our patient led to the decision to perform surgery. In April 2011, under general anesthesia and with the patient in the prone position, a laminectomy was performed from L-3 to L-5. After the exposure, the anatomy was obvious and demonstrated a bifid dura from L-3 with a bony spur at L-4, anteriorly attached to the vertebral bodies. The bony spicule was removed totally down to the level of the vertebral bodies. The dura was then opened around the defect to reveal the bifid spinal cord. The spinal cord appeared to be split from L-3 downward. The lysis of the adhesions between pia arachnoid and the dura was performed medially and laterally. The conus level was found at the level of L-5 vertebral body and the filum terminale terminated at S-2 level and spinal cord detethering was conducted. The dura was then closed posteriorly. Two months postoperative mid-sagittal, and axial T2-weighted MRI images showed removal of the bony spur and release of the spinal cord (Figure 3).

Postoperatively, the patient did not show new neurologic deficit and the treatment continued with physiotherapy. In the final follow-up we found a relative improvement of sensory and power in the right lower extremity and also in patient’s gait. He returned to work 4 months after surgery.

## 3. Discussion

Diastematomyelia is rarely diagnosed in adults. In 1990, Russel et al. (3) reported 45 adult cases with diastematomyelia introduced in the literature. We updated this search between June 1990 and August 2011, and could find only 12 more such cases, in the English literature (4, 5). Out of all reported patients (57 patients) only 10 patients (18%) were older than 50 years and only 4(%7) patients were male. A fifth male patient with diastematomyelia and age greater than 50 years is reported in our report.MRI is the diagnostic method of choice to show the entire cord and the hemicords in the vicinity of the cleft (6). CT and Metrizamide CT are the imaging methods of choice in detection of the bony spur, duplication of the dural tube and the spinal cord (6). In the present case, MRI demonstrated a combination of low laying conus (tethered spinal cord) as well as diastematomyelia and CT images showed bony spur and block vertebra.Diastematomyelia is usually associated with other spinal anomalies: spina bifida, hemivertebrae, kyphoscoliosis. Scoliosis appears in more than 50% of cases with diastematomyelia (7), females have been reported in various series to show scoliosis two to four times more often than males. Our case had spina bifida and block vertebra.It is interesting that in our case symptoms were delayed and the disease was discovered in adulthood. It might be theorised that this patient was asymptomatic until the onset of neurological difficulty.

In majority of cases, diastematomyelia is caused by a spur of bone, fibrous tissue or fibrocartilage (3), as in our patient. The neurological disturbances produced by deformity of the cord vary greatly from individual to individual. Most frequently there is difficulty in walking, caused by a weakness or paralysis of the muscles in the legs (3), as our patient presented. Sphincter disturbances of the bladder and rectum are present in about 50% of cases (3).

The treatment of adulthood diastematomyelia remains controversial due to incomplete history among patients.8 Some authors argue that the surgery should be performed only in case of progressive neurological symptoms to avoid possible complications and perioperative morbidity in still asymptomatic patients (8). We agree that the surgery in asymptomatic patients should be delayed. The clinical consequences of tethering of the cord and the deteriorating condition of our patient led to the decision to perform the surgery.

## 4. Conclusion

This report describes an adult case with the unusual etiology of a split cord malformation and diastometamyelia. Our case was unique because of the absence of any pain, neurologic signs, and precipitating acute event leading to diagnosis, until 53 years of age. Surgical decompression of bony spur provided relative improvement of his symptoms.
